# Bacteriophages as Antibacterial Agents Against Bovine Pathobionts Associated with Foodborne Human Morbidity

**DOI:** 10.3390/v18030392

**Published:** 2026-03-20

**Authors:** Mary Garvey

**Affiliations:** Department of Pharmacy, Atlantic Technological University, Ash Lane, F91 YW50 Sligo, Ireland; mary.garvey@atu.ie; Tel.: +353-071-9305529

**Keywords:** phages, resistance, ESKAPE, pathobionts, morbidity

## Abstract

Rates of foodborne infectious disease are increasing globally. The One Health zoonoses report shows increasing cases of shigatoxigenic *Escherichia coli*, campylobacteriosis, salmonellosis and listeriosis in the last 5 years. The ESKAPE pathogens are the top priority due to their alarming rate of resistance to broad-spectrum beta-lactams, carbapenems, glycopeptides, fluoroquinolones, aminoglycosides and biocide solutions. Research assessing alternative biocontrol options highlight the advantages of bacteriophages in the control of resistant bacterial species. Phage formulations including ListShield^TM^ and SalmoFresh^TM^ have gained FDA approval for food production. As biocontrol agents, however, phages are limited by their specificity in a multispecies environment, the presence of environmental variables and bacterial resistance mechanisms. Genetic modification and the use of phage cocktails aim to overcome such limitations. Future research is warranted in a harmonised approach supported by a defined legal framework to establish best formulation and exposure protocols. This review discusses phages as biocontrol agents in the control of high-risk pathobionts associated with foodborne illness. Pathobionts associated with bovine livestock are discussed due to the morbidity and incidence of disease associated with such pathogens.

## 1. Introduction

The Foodborne Disease Burden Epidemiology Reference Group (FERG) established by the World Health Organisation (WHO) estimates that, globally, there are ca. 600 million cases of foodborne disease and 420,000 associated deaths annually. Children under 5 years account for 30% of foodborne deaths [1]. The widespread presence of antimicrobial and biocidal-resistant pathogens in dairy food production highlights the need for alternative methods of preventing foodborne illness. Many zoonotic bacterial species are frequently associated with foodborne morbidity and mortality, including *Listeria monocytogenes*, *Clostridium difficile*, and many of the ESKAPE pathogens (*Enterococcus faecium*, *Staphylococcus aureus*, *Klebsiella pneumoniae*, *Acinetobacter baumannii*, *Pseudomonas aeruginosa*, *Enterobacter* spp.), which demonstrate alarming rates of antimicrobial resistance (AMR) and pan-drug resistance (PDR), demonstrating no susceptibility to any of the antimicrobial categories [2]. The WHO states that the current repertoire of antibiotics used for the treatment of ESKAPE pathogens, including broad-spectrum beta-lactams, carbapenems, glycopeptides, fluoroquinolones and aminoglycosides, are no longer demonstrating sufficient efficacy in vivo [3]. Carbapenem-resistant *K. pneumonia* and cephalosporin-resistant *Escherichia coli* moved to top priority on the WHO bacteria priority pathogen (BPPL) list in 2024. Both these strains contain the OmpA gene and efflux pump genes, e.g., small multidrug resistance (SMR) genes and tetA, tetB, tetD, and tetW, which confer tetracycline resistance [2], which confers MDR. Furthermore, *K. pneumoniae* is therapeutically challenging due to its ability to produce a capsule conferring AMR and immune resistance [4]. MDR *K. pneumoniae* is responsible for more than 69,000 deaths per year, globally [5]. *E. faecium*, having high levels of intrinsic AMR and resistance to vancomycin, is causative of MDR hospital-acquired infections (HAIs), including bloodstream infections (BSIs), urinary tract infections (UTIs) and surgical site infections (SSIs) [6]. ESKAPE pathogen resistance mechanisms include modifications in drug binding sites, active efflux pumps, porin loss, enzymatic inactivation of antibiotics and horizontal gene transfer (HGT) [7]. Efflux pumps are upregulated in the presence of sub-toxic concentrations of antibiotics and biocides; for example, marA and soxS efflux genes are upregulated in the presence of sub-toxic concentrations of ciprofloxacin [8].

Many of the ESKAPE species are common dairy pathobionts due to their enteric commensal relationship with livestock animals and excretion into the surrounding environment [9]. Extended spectrum beta lactamase-producing (ESBL) *E*. *coli*, for example, is commonly associated with resistant bovine disease [10]. *Clostridium* species are Gram-positive anaerobic pathogens associated with livestock animals and foodborne illness, e.g., as an enteric species, *C. difficile* is associated with foodborne transmission via the faecal–oral route following ingestion of contaminated foods [11], where studies have detected the presence of *C. difficile* in dairy herds [12]. AMR is proliferated in these species by the sharing of antibiotic-resistant genes (ARGs) on mobile genetic elements, such as insertion sequences, plasmids, integrons, transposons, and phage-related elements via HGT, where the excessive application of antibiotics in food-producing animals has proliferated the issue at a global level. According to the European Food Safety Authority (EFSA) and the European Centre for Disease Prevention and Control (ECDC) zoonoses monitoring and surveillance data from 2024, there is an increasing prevalence of zoonotic campylobacteriosis, salmonellosis, listeriosis and Shiga toxin-producing *E. coli* (STEC) infections (Table 1) [13]. The Centre of Disease Control (CDC) states that reducing the incidence of foodborne and enteric diseases is a public health priority [14].

While infectious disease is a significant issue with these foodborne pathogens, their role in more chronic and life-threatening conditions such as autoimmune disease and cancer must also be considered. Bovine pathobiont species *C. difficile* and *E. coli* have been described in the aetiology of colorectal cancer (CRC) and autoimmune diseases, including inflammatory bowel disease (IBD) [15]. Specifically, a correlation between *C. difficile* infection and CRC was observed in ca. 40% of patients with colibactin produced by enterotoxigenic *E. coli* associated with CRC tumorigenesis in experimental mouse models [16]. The One Health framework aims to protect human health and reduce microbial pathogenesis by applying effective control and antibiotic stewardship protocols in animal, planet and human welfare [17]. One Health recognises the need for alternative control measures to prevent and treat AMR species in human and animal therapy. The application of bacteriophage (phage) viruses which selectively infect and kill bacterial species demonstrates remarkable potential. The European Medicine’s Agency (EMA) supports phage innovation and seeks established guidelines in the production of phage-based therapies [18]. Within the dairy industry, phages have emerged as both potentially beneficial and problematic microbial species [19]. This review aims to highlight the cytotoxic potential of phages specific for bovine pathobiont species often associated with dairy food consumption. The application of phages in the biocontrol of such species may prevent foodborne infectious disease and the incidence of human cases of infectious disease, autoimmunity and CRC.

**Table 1:** Bovine pathobionts relevant to dairy foodborne illness, toxin virulence factors and MDR profiles.

Gram	Pathobiont	Toxins Present	Therapeutic Failures	Area Detected
Gram-positive	*S. aureus* CoNS [20]	Enterotoxins [21] seg, sei, sem, sen, seo and selu gene cluster on genomic island vSaβ [22]	Cefoxitin, amoxicillin, penicillin, chloramphenicol, nalidixic acid, tetracycline [20], and MRSA [10]	Food animal production and processing lines in Ethiopia [20], bovine mastitis in Ireland [10]
	*S. aureus* CoPS [20]			
	*Enterococcus* species	Strains of *E. faecium* produce BoNT/En, a botulinum neurotoxin (BoNT)-like toxin [5]	Intrinsic resistance to cephalosporins, clindamycin, aminoglycosides, and trimethoprim–sulfamethoxazole, *E. faecium* species are 80% vancomycin-resistant and 90% ampicillin-resistant [23]	Artisanal cheeses in Belgium [23]
	*C. perfringens* *C. difficile*	Alpha toxin (CPA), e.g., phospholipase C, lecithinase Toxins A (enterotoxin) and B (potent cytotoxin) C. difficile influences colonic tumorigenesis	Aminoglycosides, lincomycin, tetracyclines, erythromycin, clindamycin, penicillin, cephalosporins, fluoroquinolones	Calf and cattle livestock, Pennsylvania, Maryland and Delaware, USA [12], dairy farms, Holland
	*L. monocytogenes*	Listeriolysin O (LLO), toxin–antitoxin (TA) systems	Clindamycin, nalidixic acid, penicillin and ampicillin [24]	Humans, animals, and dairy products, Egypt [24]
Gram-negative	*K. pneumoniae*	Lipopolysaccharide (LPS) endotoxin, Colibactin Colibactin with polyketide synthesis (pks) genomic islands, cytolethal distending toxins, LPS	Carbapenem, third-generation cephalosporin [10], β-lactam antibiotics [25]	Dairy products in Libya [25]
	*E. coli*			Pathogens of bovine mastitis in Ireland [10]
	*A. baumanaii*	Toxin–antitoxin (TA) systems, e.g., higBA and mazEF [26], LPS endotoxin	Penicillin, 3^rd^-generation cephalosporins, macrolides (erythromycin) [10], β-lactam antibiotics [25], carbapenem [3]	Pathogens of Bovine Mastitis Ireland [10]
	*Salmonella* spp.	Typhoid toxin, AvrA protein, LPS endotoxin	Tetracyclines, β-lactams, aminoglycosides, fluoroquinolones, and third- and fourth-line antibiotics colistin and carbapenems	Global foodborne pathogen having MDR [13]
	*Campylobacter (C. jejuni*, *C. fetus)*	Cytolethal distending toxins, genes cdtA, cdtB, and cdtC, LPS endotoxin	Macrolides, fluoroquinolones, beta-lactams, and tetracycline	

## 2. MDR Foodborne Bovine Pathobionts

Bovine pathobionts associated with foodborne illness include microbial species present in the cow udder and intestinal system with dairy produce and meat serving as routes of transmission. In the bovine udder species, including *Staphylococcus*, pathogens categorised as coagulase-positive staphylococci (CoPS) are present [20], e.g., *S. aureus* and methicillin-resistant *S. aureus* (MRSA) and coagulase-negative staphylococci (CoNS), e.g., *S. haemolyticus*, and are associated with mastitis having varying levels of severity and treatment resistance [27]. *Enterococcus* species, including *E. faecalis*, *E faecium*, and *E. hirae,* are associated with bovine mastitis, where colonisation of the gastrointestinal tract (GIT) is associated with severe infections where translocation over the intestinal wall allows for systemic disease [23]. Similarly, *Streptococcus dysgalactiae*, *S. agalactiae* and *S. uberis* are enteric species and members of the bovine microbiota and are aetiological agents of bovine mastitis [28]. *E coli* is causative of ca. 80% of mastitis cases as an environmental pathogen; its presence in the bovine enteric system is associated with udder infection [12]. The bovine enteropathogenic *K. pneumoniae* is associated with clinical and asymptomatic mastitis in dairy cattle and dairy foodborne disease [25].

### 2.1. Antibiotic Resistance in Pathobionts

Colistin resistance in carbapenem-resistant *Enterobacteriaceae* species, including *E. coli*, has been identified clinically and in dairy herds due to the presence of the mcr-1 gene, reducing the efficacy of this last-resort antibiotic [29]. The emergence and proliferation of plasmid-mediated colistin resistance is promoted by the increased use and misuse of colistin in livestock animals [30]. *Salmonella* spp., *Listeria monocytogenes*, *Campylobacter* spp., and *C. difficile* are also prevalent agents of infectious diarrhoea in humans and livestock as entero-pathogens [31]. *C. perfringens* is a foodborne enteric pathogen causing ca. 1 million cases of foodborne illness globally, having a 30-day BSI mortality of ca. 44% [15]. Resistance to fluoroquinolone and erythromycin in *C. jejuni* isolates is rapidly increasing due to clinical and growth-promoting use in livestock animals [32]. *Salmonella* infections are associated with millions of cases of foodborne illness yearly, with the WHO listing *Salmonella* as a top 5 food-relevant organism [33]. Non-typhoidal *Salmonella* species are associated with gastroenteritis, with typhoidal *Salmonella* causing systemic, severe and often fatal cases of disease [34]. MDR *A. baumannii* is a difficult-to-treat human pathogen and is an emerging contaminant of animal food produce such as raw meat [35]. *A. baumannii*’s resistance is due to active antimicrobial enzymes, the overexpression of efflux pumps, iron acquisition, biofilm formation, motility, capsule formation, and genetic plasticity, promoting cell adhesion and tissue damage [36]. *A. baumannii* produces many β-lactamases (e.g., OXA-type carbapenemases, AmpC cephalosporinases) which hydrolyse carbapenem antibiotics, conferring resistance [37]. Studies isolated the ARG vanA gene in 96% of *E. faecalis* isolates from dairy herds having subclinical mastitis [38]. Notably, Enterococci species act as reservoirs and suppliers of mobile genetic elements (MBEs), carrying ARGs to both Gram-positive and Gram-negative species with their enteric nature allowing access [6]. MDR is prevalent in ESKAPE pathogens and *Clostridium* via the acquisition of ARGs giving resistance to oxazolidinones, lipopeptides, macrolides, fluoroquinolones, tetracyclines, beta-lactams, and their inhibitor formulations, and the WHO last-resort carbapenems, glycopeptides, and polymyxins [39]. The HGT of *mcr* genes (mcr-1 to mcr-10) via plasmids between species has proliferated colistin resistance in *E. coli*, *K. pneumoniae* and additional *Enterobacteriaceae* species [29]. *C. perfringens* has high genetic plasticity and can exchange ARGs with several bacterial species, particularly *Staphylococcus* and *Streptococcus* species [40]. Environmental pollution and foodborne transmission of MGEs and ARGs is considered biotic pollution, which represents a public health risk [2]. An excellent review of the presence of MDR ESKAPE pathogens in food sources is given elsewhere [39].

### 2.2. Bacterial Toxins and Human Morbidity

Importantly, toxins produced by such species are a health risk due to their resilience and persistence within milk processing facilities as heat-labile contaminants (Table 2). For example, Shiga toxin-encoding enterohemorrhagic *E. coli* O157:H7 is a common cause of food poisoning worldwide [33]. STEC infection is associated with undercooked beef, unpasteurized milk, and faecal-contaminated vegetables and fruit or water [41]. Staphylococcal enterotoxins are associated with high rates of food outbreaks [10]. Certain bacterial species can produce genotoxins which cause DNA mutations such as strand breaks and may be co-factors in the aetiology of CRC, cytolethal distending toxin (CDT) produced by Gram-negative bacteria, typhoid toxin produced by *Salmonella enterica* serovar *Typhi* (*S. Typhi*), and colibactin produced by certain *E. coli* and *Klebsiella*, all of which are recognised [42]. CDT is produced by various species of *Campylobacter*, *Escherichia* and *Salmonella* and induces DNA damage, cell-cycle arrest exhibits and tumorigenesis in pre-clinical models of CRC [43]. Infectious disease is an important participant in the aetiology of cancer with greater than 16% of cancer associated with microbial pathogens [44]. The chronic inflammation present in prolonged infectious disease promotes oncogenic activity in vivo, leading to disrupted cell signalling, cell proliferation, cell-cycle progression, and DNA repair alterations, leading to tumorigenesis [45]. Studies describe the increased prevalence of colibactin genes *cdt* and *pks* in *E. coli* isolated from the GIT of IBD patients having CRC compared to the healthy control group [44]. Furthermore, studies report a poor prognosis outcome and poor response to chemotherapy in CRC patients colonised by colibactin-producing *Escherichia coli* (CoPEC), which is detected in ca. 50–60% of human CRC biopsies [46]. Furthermore, the co-occurrence of fungal toxins, i.e., mycotoxins and bacterial toxins in food, is a food safety issue, as such toxins may have a synergistic effect towards cytotoxicity and pro-inflammatory responses [47].

Importantly, genes coding bacterial toxins are often present on MBEs, such as plasmids, genomic islands and prophages, as seen with enterotoxigenic *S. aureus* [21], allowing for HGT between species. Studies report the production of a botulinum neurotoxin (BoNT)-like toxin by *E. faecium* isolated from cow faeces [6]. Recent studies suggest that the potent pathogenic ability of *A. baumannii* may relate to the presence of a toxin–antitoxin (TA) system which confers a virulence factor, such as including genetic element maintenance, stress resistance, and phage inhibition [26]. Recent studies aim to determine the role of TA systems in *L. monocytogenes*, allowing stress adaptation and persistence food processing facilities [48]. The presence of lipopolysaccharide (LPS) endotoxin in Gram-negative bacteria remains a challenge clinically. LPS is released from Gram-negative cell membranes due to cytotoxicity and is released into the surrounding environment. Clinically, it is associated with cardiovascular failure, pro-inflammatory responses, organ failure and sepsis in patients [49]. LPS induces pro-inflammatory cytokines, chemokines, modified cyclooxygenase-2 (COX-2) expression and the release of prostaglandins, leading to inflammation [50]. Studies have described the pro-inflammatory role of LPS in endometriosis due to the macrophage secretion of cytokines IL-6, TNF-α, growth factors VEGF and hepatocyte growth factors in the pelvic and peritoneal areas in patients [42]. Furthermore, modifications in the LPS in cell membranes is associated with colistin resistance in Gram-negative species [37]. Polymyxins, including colistin and polymyxin-B, are last-resort antibiotics for the treatment of MDR Gram-negative bacteria [29].

**Table 2:** Role of bacterial toxins produced by foodborne bovine pathobionts in human morbidity.

Bacterial Toxin	Mode of Action In Vivo	Associated Morbidity
Cytolethal distending toxin (CDT)	Genotoxin causing double- and single-strand breaks, cell senescence, apoptosis, genomic instability, resulting in tumour initiation and progression	Colorectal cancer progression [15]
Shiga toxin	Inhibition of protein synthesis in cells by cleavage of 28S ribosomal RNA	Hemorrhagic colitis, hemolytic uremic syndrome (HUS), thrombocytopenia, and acute renal failure [41]
Enterotoxins	Increased paracellular permeability, epithelial cell damage and associated intestinal permeability, pro-inflammatory response [47]	Gastrointestinal symptoms including nausea, vomiting and diarrhoea, chronic exposure may induce autoimmunity, e.g., IBD, celiac disease, non-gastrointestinal diseases, type 2 diabetes, multiple sclerosis, and Parkinson’s disease [47]
Typhoid toxin	Cytotoxic effects via Dnase-I-like nuclease activity, induces DNA damage, resulting in cell-cycle arrest and apoptosis [51]	Typhoid fever—fever, weight loss, decrease in peripheral leucocyte count, increase in levels of pro-inflammatory cytokines (Il-6, TNF-α) [51]
Colibactin	Alteration of p53 SUMOylation and double-strand breaks, generates DNA adducts [46]	Colorectal cancer progression, disease recurrence, resistance to treatment, morbidity [46]
Toxin–antitoxin (TA) systems	Promoting plasmid maintenance, slow growth and dormancy in bacterial cells	Proliferation of antibiotic resistance, MDR and bacterial persistence in infectious disease of ESKAPE pathogens and TB
Endotoxin–LPS toxin	Biosynthesis of major inflammatory mediators, e.g., TNF-α, IL1-β and IL-6, pyroptosis and activation of the NLRP3 inflammasome followed by secretion of IL-1β and IL-18 [49,50]	Sepsis, multiple organ dysfunction [49], autoimmunity, LPS from *E. coli* induces a more potent immune response than other endotoxins, endometriosis [42]

## 3. Bacteriophages Against Foodborne Pathobionts

Bacteriophages (phages) are obligate intracellular parasites of bacteria which selectively infect and reproduce within bacterial species and are the most abundant species on earth. As viruses which selectively infect bacteria, lytic phages have long been recognised for their potent antibacterial activity resulting from cell lysis, i.e., bactericidal action [52]. A review of phages and their enzymes is provided elsewhere [53]. Phage therapy is currently provided in Belgium for chronic rhinosinusitis, pulmonary infections such as bronchiectasis and cystic fibrosis, musculoskeletal infections and sepsis, as assessed by the Coordination group for Bacteriophage Therapgy Leuven (CBL) (UZ Belgium). There are three phage families having biocidal activity against ESKAPE pathogens, namely, *Myoviridae*, *Podoviridae*, *and Siphoviridae* [7]. It must be noted, however, that the International Committee on Taxonomy of Viruses (ICTV) has abolished these morphology-based identities and the order *Caudovirales*, and has established a new framework which classifies phages based on genomic similarity and core gene phylogenies into the class *Caudoviricetes* [54].

The antibacterial activity of phages and their endolysins against ESKAPE pathogens and *Campylobacter* and *Salmonella* have been studied where efficacy has been demonstrated [52]. A bacteriophage cocktail successfully inactivated foodborne *S. enteritidis* and *S. typhimurium* on chicken meat [55]. For example, the phage EF-M80 targeting *E. faecium* phage has demonstrated antibacterial and anti-biofilm efficacy, with additional *E. faecium* siphovirus-type phages (e.g., EfV12-phi1) and myoviruses (e.g., MDA2, Porthos, iF6) also having efficacy against VRE strains [56]. Studies describe a 2.8 log_10_ CFU reduction in *S. aureus* isolated from raw meat using phage mSA4 after 24 h at 4 °C [57], with the polyvalent phage PS5 having activity against *S. enteritidis*, *S. typhimurium*, and *E. coli* O157: H7 in beef samples [54]. *S. aureus* isolated from milk samples proved sensitive to phage Vb-SAV-SDQ, with milk-isolated *S. enteritidis*, *S. typhimurium*, and *E. coli* O157: H7 having sensitivity to polyvalent phage PS5 [58]. The studies of Ferriol-González et al. (2024) investigated the efficacy of phage cocktails against MDR *Klebsiella* species, including 58 carbapenem-resistant clinical isolates and capsulated *K. pneumoniae* strains [5]. A phage cocktail against *K. pneumoniae* decreased gut inflammation in a mouse IBD model without altering the intestinal microbiome [40].

The review of Venhorst et al. (2022) describes the efficacy of phages and endolyisns against *C. perfringens* and *C. difficile* [59]. The CPD2 phage of *C. perfringens* was isolated from chicken meat, where its endolysin LysCPD2 demonstrated efficacy against *C. perfringens*, *B. cereus* and *B. subtilis* [60]. The *Campylobacter* phage CC_R7 demonstrated significant lytic activity against *C. jejuni* planktonic cells and biofilms isolated from poultry meat [61]. The studies of Calancha-Padrón et al. (2026) isolated three novel lytic bacteriophages with activity against fluoroquinolone-resistant *Campylobacter* isolates, including *C. jejuni* [62]. The bacteriophage Phab24 has efficacy against colistin-resistant *A. baumannii*, with a nine-phage cocktail effectively treating an MDR *A. baumannii* infection in a 68-year-old diabetes patient with necrotizing pancreatitis [36]. The studies of Meile et al. (2023) developed a rapid bacteriophage diagnostic assay which detected *E coli*, *Enterococcus* spp., and *Klebsiella* spp. in cases of urinary tract infections [63].

The efficacy of numerous phages against the biofilms of many foodborne pathobionts has also been demonstrated, including species *L. monocytogenes* and STEC [58]. Augmenting the phage pB3074 with cefotaxime and meropenem effectively eradicated *A. baumannii* biofilms in animal disease models [36]. The phage cocktail BEC8100 eradicated *E. coli* biofilms from food processing surfaces, including stainless steel, ceramic tile, and polyethylene, with phage P100 disrupting biofilm formation by *L. monocytogenes* on stainless steel [64]. Microbial biofilms are problematic for the food industry, as their presence allows for microbial persistence post-disinfection protocols on abiotic and biotic surfaces. Biofilms are the natural viable state of bacterial species, including foodborne pathogens *L. monocytogenes*, *A. baumannii*, *Clostridium*, *Streptococcus species*, *S. aureus*, *Salmonella* spp., and pathogenic *E. coli* [58]. Biofilms are associated with ca. 60% of foodborne disease outbreaks [65]. Current FDA-approved phage cocktails for food production include ListShield^TM^ against *L. monocytogenes*, Listex P100, EcoShield^TM^ against *E. coli O157:H7*, SalmoFresh^TM^ against *Salmonella enterica* and ShigaShield^TM^ against *Shigella*, which are Generally Recognised as Safe (GRAS) for use in food production [66]. The Electronic Code of Federal Regulations (eCFR-FDA) lists *L. monocytogenes* phage formulation as a food additive according to good manufacturing practice (GMP), where it is sprayed directly onto ready-to-eat meat products in the control of *Listeria* contamination [64]. Antimicrobial agents’ phages have many advantages (Table 3), especially when applied as phage cocktails or as combined therapy with antibiotics. Phage enzymes, including endolysins and peptidoglycan hydrolases (termed enzybiotics), which are released by phages to lyse bacterial cells, may be used in conjunction with phage preparation, antimicrobial peptides or antibiotics. The endolysin LysH5 demonstrated efficacy against *S. aureus*, which was increased in combination with the bacteriocin nisin in milk [67].

### 3.1. Barriers to Implementation in the Food Industry

In food production facilities, lytic phages may be implemented as disease treatment and biocontrol options (Table 3). In dairy production, *Lactococcus lactis* phages are recognised as influencers of the fermentation process and as contaminants of milk produce. Phages can negatively impact food production due to reduced yield, product losses, economic losses, raw material waste, reduced product quality and inappropriate microbial proliferation [64]. Alternatively, they may offer therapeutic aid in the treatment of mastitis, respiratory disease, metritis and lameness in dairy herds, improving animal welfare according to One Health [68]. For example, cases of mastitis resulting from *S. aureus* have been treated with phage therapy, with phages also successfully removing *Staphylococcus* biofilms [52]. Isolating and identifying suitable lytic phages against foodborne pathogens remains a challenge. Relevant phages may be found in environmental reservoirs such as wastewater and sewage. The studies of Tian et al. (2024) isolated the Abgy202141 phage from sewage, which is selective for *A. baumannii* and does not carry any AMR or virulence genes [69].

Phage resistance remains a challenge in phage therapy, as bacterial species manifest resistance via alteration or elimination of the phage-binding receptors, preventing attachment, blocking the insertion of phage nucleic acids and the use of restriction–modification enzymes or the CRISPR-Cas adaptive immune system [70]. Bacterial species also have a toxin–antitoxin system, producing a toxin which stops vital biochemical reactions arresting cell growth, thus preventing phage reproduction; the antitoxin then deactivates this toxin [26,48]. The capsule of *Klebsiella* sp. hinders phage infection and reproduction, allowing for bacterial survival [5]. The application of phage cocktails and augmentation with antibiotics offers the potential to overcome such resistance methods. Developing effective targeted phage cocktails, however, requires a detailed understanding of phage–bacterial receptor interactions, which is currently lacking [71].

Phage specificity may be a limiting factor in facilities such as slaughterhouses and milking parlours, where several contaminating microbial species and organic matter are present [72]. The nucleic acid content of phages renders them susceptible to water, temperature, UV and chemical degradation, impacting stability and efficacy [19]. Additionally, the efficacy of phages against persister cells, slow-growing variant cells and bacterial endospores remains unknown [73].

Phage formulation is an important factor when developing phage therapeutics and biocides. Enteral phage delivery for gastrointestinal disease is problematic, as the acidic gastric environment may destroy phages [74]. Formulations under investigation for phage delivery include encapsulation in liposomes or hydrogels, freeze drying, spray drying, phage suspensions, electrospinning nanostructures [68] and aerosol delivery formulations for respiratory disease [64]. The application of enzybiotics in food production is hindered by the environmental variations in processing facilities, i.e., cold and high temperatures, high acidity, and food matrix, e.g., sugar and fats, which impact enzyme efficacy [52].

Currently, there is a lack of definite legislation and approval frameworks for the use of phages in food production. The FDA’s GRAS recognition permits safe substances to be used in food production and in animal feed without pre-market approval [66]. The European Food Safety Authority (EFSA) has not approved phage products for food production, which hinders the use of phages in the EU. The EMA, however, which supports phage innovation and seeks established guidelines in the production of phage-based therapies [18], has reported the possible applications of phages through the guidelines on the evaluation of medicinal products indicated for the treatment of bacterial infection [18].

**Table 3:** Outline of the advantages, disadvantages and limitations relating to the use of phages in food production facilities.

Advantages	Disadvantages	Limitations
Bacterial specificity.	Limit therapeutic application due to specificity.	Isolation and identification of species-specific phages is problematic.
Self-amplifying effect or self-replicating.	Bacterial resistance to phages limits efficacy [70].	Absence of harmonisation in production and regulatory frameworks [75].
Application of phage cocktails and/or enzyme mixes (enzybiotics).	Transmission of phage-encoded toxins, e.g., diphtheria toxin, cholera toxin, Shiga toxin [76] and pathogenicity islands.	Inhibitory food matrix or presence of organic matter.
Colourless and tasteless, not impacting the organoleptic quality of food [58].	Sensitive to denaturation and degradation, impacting stability and activity.	Storage issues at farm level as prone to UV degradation [52].
Biocompatible with animals and humans.	Phage endolysins are effective against Gram-positive species but are hindered by the outer membrane of Gram-negative species [52].	*C. difficile* phages are temperate phages, lytic phages are required for biocontrol strategies.
Green—non-environmental pollutants—GRAS recognition [66].	Phage and prophage carriage and HGT of AMR genes.	Phages are not stable at higher temperatures.
May act as diagnostic aids [75].	Potential immune response in vivo, which aims to eliminate phages [77].	Capsule confers resistance to certain species, including *Klebsiella* [5].
Amenable to genetic modification [8].		The rapid lysis of the bacterial cell may release endotoxin LPS and antigens, which induce sepsis [77].

### 3.2. Phages and Prophages in Gut Health and Morbidity

Phages and prophages, which are viral genomes integrated into the host chromosomes, act as carriers and suppliers of bacterial toxin genes, which may have clinical consequences. For example, prophages constitute ca. 20% of the bacterial genome. Bacterial toxins coded on phage genomes include the Shiga toxin in *Shigella* sp., the cholerae toxin in *Vibrio* sp., the diphtheria toxin in *Corynebacterium*, the botulinum toxin in *C. botulinum* and the binary toxin of *C. difficile* [78,79]. Phage and prophage HGT is also associated with the transmission of AMR genes, which alter the host physiology and impart antibiotic resistance and virulence [40]. Phages are associated with the transfer of the mecA gene, conferring methicillin resistance in *Staphylococcus,* tet genes in *S. Typhimurium*, and efflux pump genes [75], which also promote biocidal resistance in bacterial species [49]. Studies detected *bla*_TEM_, *bla*_CTX-M-1_, *mecA*, *armA*, *qnrA*, and *qnrS*, *bla*_TEM_, *qnrA*, and *bla*_CTX-M-1_ resistance genes on phages isolated from faeces [80]. Therefore, phages can promote virulence and pathogenicity in bacterial species, as they improve bacterial fitness, ultimately impacting the animal host [81]. Studies show that prophages carried ca. 5.8% and 2.5% of toxin-encoding and AMR genes, respectively [76]. Studies have identified prophages in many pathogens, including the pathobionts *C. difficile*, *S. enterica*, *K. pneumoniae*, and *Helicobacter pylori*, amongst others [76]. The *sopE* gene carried on a prophage increased *S. Typhimurium* growth in vivo [82]. Prophages carry Shiga toxin genes (*stx)* in *E. coli* O157:H7, which are causative of haemorrhagic colitis and haemolytic uremic syndrome (HUS), associated with endothelial cell cytotoxicity [83]. Enterotoxin A, which is causative of *Staphylococcus* food poising, is carried by a bacteriophage, as are *Staphylococcal* pathogenicity islands (SaPIs), associated with toxic shock syndrome toxin, and enterotoxin B [84]. Studies describe the carriage of *C. difficile* toxin genes TcdA, TcdB and binary toxin on prophages, which were believed to be entirely chromosomal in nature [82]. Importantly, prophages are associated with toxin genes and sporulation in *C. perfringens* and impact frequency of sporulation, sporulation induction and spore germination [85], which is key to pathogenicity and survival in food production facilities. Phages present in *C. botulinum* pathogenic strains are carriers of the gene coding for BoNTs [82]. Integrated prophage genomes respond to an induction signal, which causes them to leave the host genome and begin replication [81]. Prophages carrying toxin genes can contribute to morbidity in animal species by disrupting the microbiome and epithelial gut health [15]. The activation of prophages can alter the intestinal microbiome, resulting in inflammation in animals, and is associated with IBD in humans [40]. Furthermore, the presence of lysed bacterial cells after phage reproduction may promote inflammatory response and disease progression [86]. Recent studies assessing dysbiosis in the aetiology of CRC in patients suggest a complex role, with phage involvement [87].

## 4. Future Direction of Bacteriophages

Advances in genetic engineering may aid in optimising phage specificity and may provide improved antimicrobial application. Genetically modified (GM) phages can be designed to produce an increased number of enzybiotics and suppress bacterial resistance mechanisms while maintaining phage specificity and replication capacity [8]. Genetically engineering the *E. coli* T7 phage to produce the hydrolase enzyme improved biofilm penetration and degradation and provided a 99% elimination of the biofilm [72]. The overexpression of efflux pumps is prominent in MDR ESKAPE pathogens. Enhancing the phage targeting of bacterial efflux pumps via GM may improve efficacy, as pumps such as the Tolc and OprM act as phage receptors, where overexpression may increase phage susceptibility and reduce antibiotic resistance [8]. In the ESKAPE pathobionts, the efflux gene TolC in *E. coli* and *K. pneumoniae* and OmpA in *A. baumannii* are targeted by phages U136B and AB1, respectively [88]. The GM phage M13mp18 was designed to overexpress *lexA3*, which represses the DNA repair system in *E. coli*, increasing sensitivity to antibiotics [89]. The antibacterial compound LNT113 was developed by binding cecropin A with the endolysin EC340, which improved membrane permeability and increased activity and synergistic action with antibiotics [77]. The studies of Hsu et al. (2020) engineered a temperate phage to repress the Stx toxin in STEC in vivo [90]. Phages have also been engineered to prevent biofilm formation by disrupting quorum sensing and cell communication. The phage T7select415-1 was modified to produce a *Bacillus anthracis* enzyme which interferes with quorum sensing, which prevented biofilm formation [91]. Methods for engineering phages that are in use include direct cloning, homologous recombination with or without CRISPR-Cas and whole-genome activation [63]. Artificial intelligence (AI) has led to the development of AlphaFold, which predicts protein structure and models phage receptor binding, allowing for improved specificity towards resistant bacteria [92].

Bacteriophages and phage cocktails may offer diagnostic techniques for detecting and identifying bacterial pathogens in both food production facilities and clinical settings. Traditional microbial diagnostics relies on culture-based methods, nucleic acid amplification via PCR and ELISA-based antigen detection, which are reproducible, automated and adaptable to polymicrobial infections in clinical settings [75]. In food production, such methods are hindered by the food matrix and by whatever organic matter and variable species might be present, and is relatively slow to generate results. The ISO 11290-1:2017 (reviewed in 2024) guidelines for the detection of *Listeria* species in feed intended for human and animal consumption requires up to 48 h for culture followed by 96 h for morphological identification [93]. Phages can be designed as biosensors having a transducer combined to a bio receptor, which sends a biological signal which a computer recognises as a digital signal [63]. Phage proteins such as the cell wall-binding domains (CBDs) of endolysins and receptor binding proteins on tail fibres have been used to design protein-based detection towards Gram-positive (*Listeria*, *Clostridium*) and Gram-negative (*Salmonella*, *Shigella*) species, respectively [94], where a combination of both CBD and receptor proteins was used to detect *Listeria* serovars [95]. An excellent review of phage biosensors is provided elsewhere [96,97]. The isolation of new phages against ESKAPE pathobionts is needed to optimise phage cocktails. The studies of Pradal et al. (2023) isolated and characterised the phage vB_EfaH_163 using genomic methods, which has activity against a range of *E. faecium* and *E. faecalis* strains in vivo without the presence of AMR or virulence genes [98].

Recent studies highlight the advantages of phages as vaccine modalities due to their ability to induce both innate and adaptive immune responses, by inducing cytokines, inflammation, antigen-presenting cells (APCs) and immunology memory [99]. Phages can also be GM to carry specific antigens and chemical mediators of immunity, such as cytokines, e.g., interleukin (IL). The studies of Brišar et al. (2025) designed an anti-cancer phage vaccine which demonstrated efficacy as a standalone and when combined with IL-12 plasmids [100]. Cancer vaccines consist of plasmids of nucleic acid sequences coding for tumour antigenic proteins which are delivered in vivo to APCs, which subsequently activate anti-cancer cytotoxic T-cells and helper T-cells, inducing anti-cancer action [101].

## 5. Conclusions

In an era of increasing antibiotic resistance and infectious disease risk, the development of effective antimicrobial alternatives is an urgent need. The EMA considers bacteriophages as having potential as therapeutics and medical products. Bacteriophages have potent bactericidal activity, targeted specificity, and anti-biofilm action, which, when combined with their self-replicating ability, makes them desirable antimicrobial agents. Their application in food-producing facilities as biocides and potential metaphylactic agents is hindered by formulation issues, the presence of organic matter, contact time, bacterial load, biocides and temperature variations on site. Phage resistance has been identified in many species, including the ESKAPE pathobionts, which may be overcome by the use of phage cocktails and by genetically engineering phages. Phages approved by the FDA are currently applied in food production to control *L. monocytogenes* ListShield^TM^, Phageguard L, *E. coli O157:H7* EcoShield^TM^ and *Salmonella* SalmoFresh^TM^. Additionally, the FDA has approved phage preparations in cases of clinical emergencies in the EU and Australia, with clinical trials currently focusing on MDR bacteria, particularly ESKAPE pathogens. Wider applications of phages include biosensors, microbial detection and vaccine modalities, which are supported by advances in gene editing technology and AI. Overcoming the limitations of phages, such as pharmacokinetics in vivo, immune stimulation and phage carriage of undesirable genes, requires ongoing research which must be supported by harmonised methods aligned to a legal framework.

## Data Availability

No new data were created or analysed in this study.
